# Blastic plasmacytoid dendritic cell neoplasm: diagnostic difficulty and proposed flowchart for histopathological diagnosis^☆^

**DOI:** 10.1016/j.abd.2022.05.006

**Published:** 2023-03-03

**Authors:** Gabriel Taylor Castolde, Alexandre Lizardo Lourenço Pontes, Gabriel Macedo Cortopassi, José Cândido Caldeira Xavier-Júnior

**Affiliations:** a Faculty of Medicine, Centro Universitário Católico Unisalesiano Auxilium, Araçatuba, SP, Brazil; b Private Practice, Araçatuba, SP, Brazil; c Faculty of Medicine, Universidade Estadual Paulista, Botucatu, SP, Brazil; d Instituto de Patologia de Araçatuba, Araçatuba, SP, Brazil

Dear Editor,

Blastic plasmacytoid dendritic cell neoplasm (BPDCN) is a rare lymphoma/leukemia with an incidence of 0.44 cases per 100,000 people, with a mean age range of 53 to 68 years and a male-to-female ratio of 3:1.^1^, ^2^

The etiology of BPDCN is unknown. The presentation and clinical course of the disease are widely heterogeneous.^1^, ^2^ There are no documented genetic, environmental, or hereditary factors that predispose to its development, but approximately 10% to 20% of the patients have a previous history of hematological malignancies.^3^

BPDCN is characterized by aggressive behavior with rapid spread. When present, skin lesions appear as erythematous and/or violaceous plaques, papules or nodules, either single or multiple, with firm consistency, pruritic or not. It is noteworthy that BPDCN may already initially present with systemic involvement or develop, from the primary cutaneous form, into the secondary involvement of lymph nodes with systemic symptoms, such as weight loss. The mean survival comprises a few months (12 to 14 months).^1^, ^4^ Therefore, being an extremely rare disease with diverse clinical manifestations that remain scarcely described, the diagnosis is a challenge from the clinical and histopathological point of view.

In view of the above, the aim of this article is to describe a case of BPDCN, diagnosed in the early stages, focusing on the histopathological findings through the proposition of a flowchart for histopathological diagnosis.

## Case report

**Figure 1:**
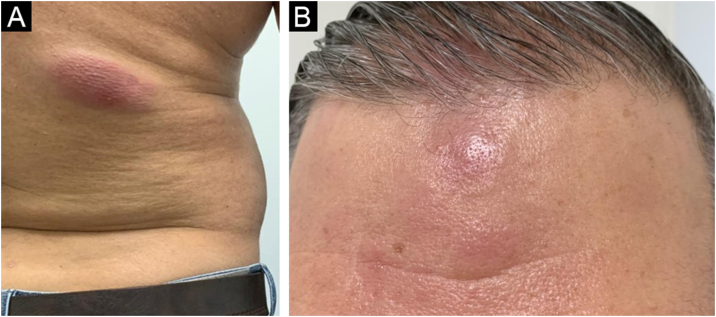
(A) Violaceous plaque on the lower dorsal region. (B) Infiltrated erythematous nodules and plaques on the forehead.

A 38-year-old male patient showed erythematous-violaceous plaques and nodules associated with pruritus and distributed over the trunk, upper limbs and forehead (Fig. 1) on dermatological clinical examination. He denied weight loss, fever, or lymph node enlargement and did not report previous important diseases. A biopsy was performed, with the clinical hypotheses of drug reaction, sarcoidosis, urticarial vasculitis and mycosis fungoides.

**Figure 2:**
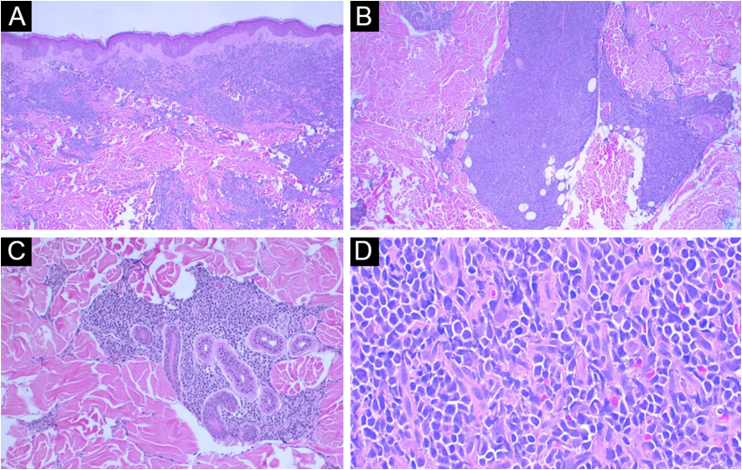
(A) Panoramic view: note the intact epidermis and the Grenz zone (Hematoxylin & eosin, ×40). (B) Note the involvement of the deep dermis (Hematoxylin & eosin, ×100). (C) In detail, area of periadnexal involvement around the sweat glands (Hematoxylin & eosin, ×200). (D) Detail of cells with immature morphology, scarce cytoplasm and granular chromatin (Hematoxylin & eosin, ×400).

**Figure 3:**
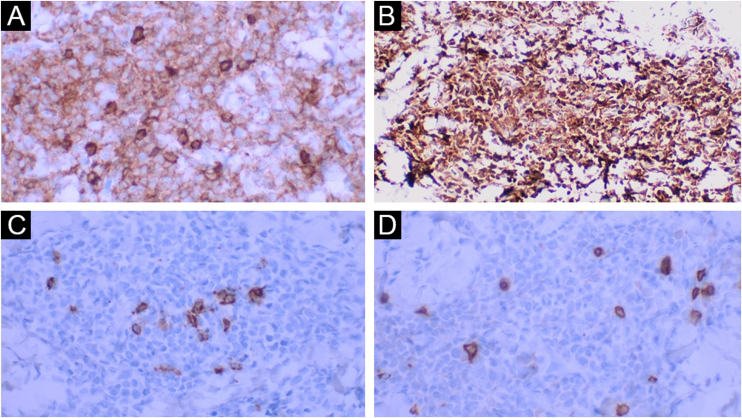
(A) Positive CD45 (×400). (B) Positive CD123 (×400). (C) Negative CD20 in the neoplasm and positive in the rare, intermingled B lymphocytes (×400). (D) Negative CD3 in the neoplasm and positive in the intermingled T lymphocytes (×400).

**Figure 4:**
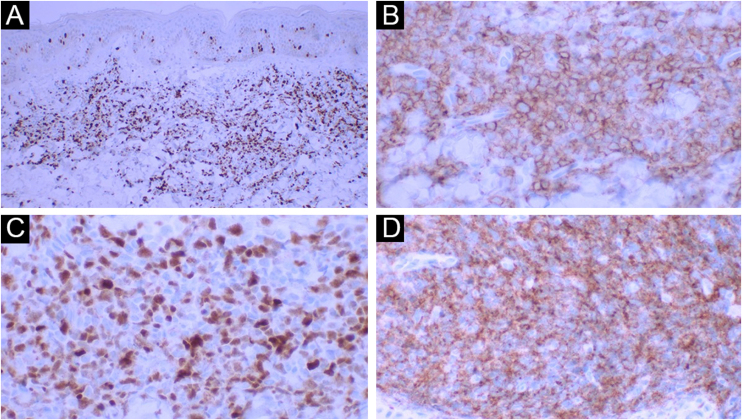
(A) Ki-67 positivity illustrating the high rate of cell proliferation (×40 – panoramic view). (B) Positive CD43 (×400). (C) Positive TDT (×400). (D) Positive CD56 (×400).

Histopathological examination disclosed a dense, superficial and deep dermal infiltrate, with diffuse and periadnexal involvement. The infiltrate was characterized by cells with blast morphology, displaying irregular nuclei, granular chromatin, one or more nucleoli, and scarce agranular cytoplasm (Fig. 2). There were frequent mitoses (more than five mitoses per 1 mm^2^), including atypical forms. Necrosis and angioinvasion were not observed. The epidermis was preserved and separated from the neoplasm by a Grenz zone. Immunohistochemistry showed positivity for CD45, CD4, CD43, CD56, TDT, CD123, a high cell proliferation index (Ki-67 estimated at 50%) and negativity for the other tested markers (CD3, CD20, CD30, CD34, CD138, and myeloperoxidase; Table 1, Figure 3, Figure 4). Complementary exams performed included computed tomography (CT) of the skull, thorax and abdomen; cerebrospinal fluid (CSF) sampling; myelogram; bone marrow karyotype; immunophenotyping of the bone marrow and bone marrow biopsy. They did not reveal extracutaneous involvement by the neoplasm.

**Table 1:** Results of the immunohistochemical panel applied in the present case.

Antibody	Clone	Result
CD3	Policlonal	Negative
CD4	4B12	Positive
CD20	L26	Negative
CD30	Ber-H2	Negative
CD34	QBEnd 10	Negative
CD43	DF-T1	Positive
CD45	2B11+PD7/26	Positive
CD56	123C3	Positive
CD68	PG-M1	Positive
CD123	6H6	Positive
CD138	MI15	Negative
TDT	EP266	Positive
Myeloperoxidase	Policlonal	Negative
Ki-67	MIB-1	Positive (High index: estimated at 50%)

The HyperCVAD scheme (hyperfractionated cyclophosphamide, vincristine, doxorubicin, and dexamethasone alternated with high doses of methotrexate and cytarabine) was the treatment used in this case, with good results, and the patient is still being evaluated for a possible allogeneic bone marrow transplant.

## Discussion

BPDCN was first described in 1994 as an NK cell lymphoma, due to the positive expression of CD56, and it was only in 2016 that it was classified as separate type of lymphoma by the World Health Organization (WHO).^1^

The poor prognosis requires early suspicion and diagnosis so that treatment can be as effective as possible. For surgical pathologists, BPDCN must be considered in case of dermal infiltration by neoplastic cells with blast morphology. Immunohistochemistry is essential for the final diagnosis.

**Figure 5:**
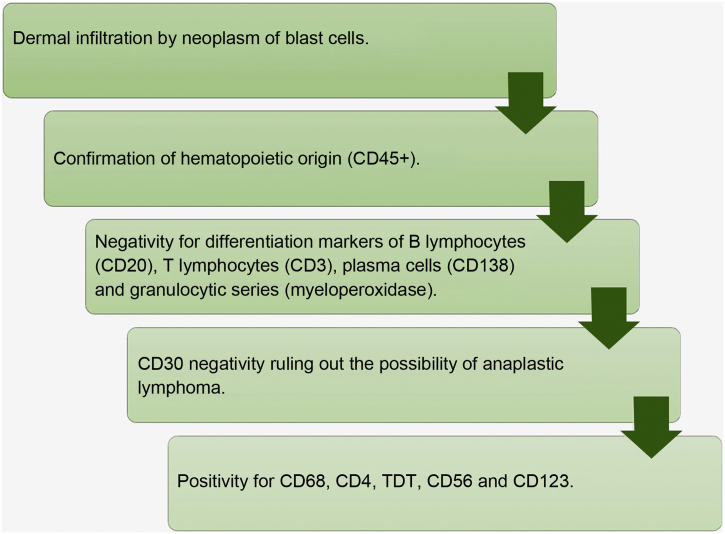
Flowchart for histopathological diagnosis of blastic plasmacytoid dendritic cell neoplasm.

In the presence of a case with dermal infiltration by a poorly differentiated neoplasm, CD45 positivity confirms the hematolymphoid origin. Negativity for CD3, CD20, CD138 and myeloperoxidase markers exclude T lymphocyte, B lymphocyte, plasma cell and myeloid differentiation, respectively. CD56 positivity confirms the cytotoxic profile. It should be noted that BPDCN also expresses T cell markers such as CD4 and CD43, as well as markers of immature cell neoplasms such as TDT; however, there is no CD34 expression, a frequently positive marker in blast cells (Fig. 5).

Despite the possible good initial response to chemotherapy, the cases invariably show recurrence, with fulminant leukemia being one of the main causes of death.^1^ The main differential diagnoses from the histopathological point of view are cutaneous infiltration by myeloproliferative neoplasms or myelodysplastic syndrome.^1^, ^5^

Considering the other CD56-positive cutaneous neoplasms, the diagnosis based on clinical aspects, microscopic morphology and immunohistochemistry, allows ruling out the other differential diagnoses.^6^ Clinically, it is possible to rule out the possibility of lymphomatoid papulosis. Moreover, CD30 negativity helps to rule out cutaneous CD30-positive neoplasms. From the morphological point of view, the absence of necrosis, angiocentrism and destruction of the vascular wall rule out the possibility of an extranasal T/NK cell lymphoma. CD56-positive acute myeloid leukemia (AML) skin infiltration may share immunophenotypic markers with BPDCN; however, AML will rarely be CD123-positive and more commonly expresses myeloperoxidase, a negative marker in BPDCN.^6^

As for treatment modalities, the impact of using anti-CD123 targeted therapy (tagraxofusp) for BPDCN remains debatable; however, studies show a good response to this drug either as the first line of treatment or for disease recurrence.^7^

It must be considered that the low incidence of BPDCN influences the small number of available therapeutic possibilities, as well as hinders a deeper understanding of this neoplasm from a molecular point of view. It is noteworthy that, contrary to the false idea that dermatological diseases have a good prognosis, BPDCN must be recognized by dermatologists and pathologists to accelerate the diagnosis and allow early treatment.

## Financial support

None declared.

## Authors' contributions

Gabriel Taylor Castolde: Literature review and writing of the manuscript.

Alexandre Lizardo Lourenco Pontes: Dermatologist responsible for patient care; performance of the biopsy and critical review of the final version of the manuscript.

Gabriel Macedo Cortopassi: Hematologist responsible for the treatment, help writing the manuscript and critical review of the final version of the manuscript.

José Cândido Caldeira Xavier-Júnior: Design of the manuscript and the flowchart; writing of the manuscript; study supervision and review of the final version of the manuscript.

## Conflicts of interest

None declared.

## References

[1] Elder D.E., Massi D., Scolyer R.A., Willemze R. (2018). WHO Classification of Skin Tumours.

[2] Venugopal S., Zhou S., El Jamal S.M., Lane A.A., Mascarenhas J. (2019). Blastic Plasmacytoid Dendritic Cell Neoplasm-Current Insights. Clin Lymphoma Myeloma Leuk..

[3] Pagano L., Valentini C.G., Pulsoni A., Fisogni S., Carluccio P., Mannelli F. (2013). Blastic plasmacytoid dendritic cell neoplasm with leukemic presentation: an Italian multicenter study. Haematologica..

[4] Winfield H.L., Smoller B.R., Bolognia J.L., Jorizzo J.L., Schaffer J.V. (2015). Dermatologia.

[5] Bénet C., Gomez A., Aguilar C., Delattre C., Vergier B., Beylot-Barry M. (2011). Histologic and immunohistologic characterization of skin localization of myeloid disorders: a study of 173 cases. Am J Clin Pathol..

[6] Assaf C., Gellrich S., Whittaker S., Robson A., Cerroni L., Massone C. (2007). CD56-positive haematological neoplasms of the skin: a multicentre study of the Cutaneous Lymphoma Project Group of the European Organisation for Research and Treatment of Cancer. J Clin Pathol..

[7] Serio B., Giudice V., D’Addona M., Guariglia R., Gorrese M., Bertolini A. (2020). A case series of blastic plasmacytoid dendritic cell neoplasia. Transl Med UniSa..

